# Host Cell Entry and Neutralization Sensitivity of SARS-CoV-2 Lineages B.1.620 and R.1

**DOI:** 10.3390/v14112475

**Published:** 2022-11-09

**Authors:** Anzhalika Sidarovich, Nadine Krüger, Cheila Rocha, Luise Graichen, Amy Kempf, Inga Nehlmeier, Martin Lier, Anne Cossmann, Metodi V. Stankov, Sebastian R. Schulz, Georg M. N. Behrens, Hans-Martin Jäck, Stefan Pöhlmann, Markus Hoffmann

**Affiliations:** 1 Infection Biology Unit, German Primate Center, 37077 Göttingen, Germany; 2 Faculty of Biology and Psychology, Georg-August-University Göttingen, 37073 Göttingen, Germany; 3 Department of Anesthesiology, University of Göttingen Medical Center, Georg-August University of Göttingen, Robert-Koch-Straße 40, 37075 Göttingen, Germany; 4 Department for Rheumatology and Immunology, Hannover Medical School, 30625 Hannover, Germany; 5 German Centre for Infection Research (DZIF), Partner Site Hannover-Braunschweig, 30625 Hannover, Germany; 6 Division of Molecular Immunology, Department of Internal Medicine 3, Friedrich-Alexander University of Erlangen-Nürnberg, 91054 Erlangen, Germany; 7 Centre for Individualized Infection Medicine (CiiM), Feodor-Lynen-Straße 7, 30625 Hannover, Germany

**Keywords:** SARS-CoV-2, spike protein, B.1.620, R.1, cell entry, neutralization, antibody evasion, ACE2 binding

## Abstract

The spike (S) protein of severe acute respiratory syndrome coronavirus 2 (SARS-CoV-2) facilitates viral entry into host cells and is the key target for neutralizing antibodies. The SARS-CoV-2 lineage B.1.620 carries fifteen mutations in the S protein and is spread in Africa, the US and Europe, while lineage R.1 harbors four mutations in S and infections were observed in several countries, particularly Japan and the US. However, the impact of the mutations in B.1.620 and R.1 S proteins on antibody-mediated neutralization and host cell entry are largely unknown. Here, we report that these mutations are compatible with robust ACE2 binding and entry into cell lines, and they markedly reduce neutralization by vaccine-induced antibodies. Our results reveal evasion of neutralizing antibodies by B.1.620 and R.1, which might have contributed to the spread of these lineages.

## 1. Introduction

The severe acute respiratory syndrome coronavirus 2 (SARS-CoV-2) is responsible for the coronavirus disease 2019 (COVID-19) pandemic. Vaccines protect against severe COVID-19, and vaccine-induced neutralizing antibodies are believed to be important for protection [1,2,3]. Furthermore, recombinant, monoclonal neutralizing antibodies are used for COVID-19 treatment [4,5]. The viral spike (S) protein employs the cellular receptor ACE2 [6,7] and an S protein-activating cellular protease (TMPRSS2 or cathepsin L) for host cell entry. Importantly, the S protein interface with ACE2 is a key target for neutralizing antibodies [8]. Mutations in the S proteins of emerging SARS-CoV-2 lineages can allow evasion of neutralizing antibodies and may alter virus–host cell interactions during viral entry, thereby potentially modulating viral transmissibility. However, the S proteins of several SARS-CoV-2 lineages remain to be analyzed for their capacity to mediate viral entry and their neutralization sensitivity. Here, we analyzed the S proteins of lineages B.1.620 and R.1.

## 2. Materials and Methods

### 2.1. Cell Culture

HEK-293T (human, female, kidney; ACC-635, DSMZ; RRID: CVCL_0063), Vero (African green monkey kidney, female, kidney; CRL-1586, ATCC; RRID: CVCL_0574, kindly provided by Andrea Maisner) and Huh-7 cells (human, male, liver; JCRB Cat# JCRB0403; RRID: CVCL_0336, kindly provided by Thomas Pietschmann) were maintained in Dulbecco’s modified Eagle medium (DMEM, PAN-Biotech, Aidenbach, Germany). Calu-3 (human, male, lung; HTB-55, ATCC; RRID: CVCL_0609, kindly provided by Stephan Ludwig) and Caco-2 cells (human, male, colon; HTB-37, ATCC, RRID: CVCL_0025) were maintained in minimum essential medium (Thermo Fisher Scientific, Waltham, MA, USA). All media were supplemented with 10% fetal bovine serum (Biochrom, Berlin, Germany) and 100 U/mL penicillin and 0.1 mg/mL streptomycin (PAA Laboratories GmbH, Cölbe, Germany). Furthermore, Calu-3 and Caco-2 cells received 1× non-essential amino acid solution (from 100× stock, PAA Laboratories GmbH) and 1 mM sodium pyruvate (Thermo Fisher Scientific). All cell lines were incubated at 37 °C in a humidified atmosphere containing 5% CO_2_. Cell lines were validated by STR-typing, amplification and sequencing of a fragment of the cytochrome c oxidase gene, and/or microscopic examination with respect to their growth characteristics. In addition, cell lines were regularly tested for mycoplasma contamination. Transfection of cells was carried out by calcium-phosphate precipitation.

### 2.2. Plasmids

Plasmids encoding DsRed, VSV-G (vesicular stomatitis virus glycoprotein), SARS-CoV-2 S B.1 (codon optimized, contains C-terminal truncation of 18 amino acid), SARS-CoV-2 S B.1.617.2, and soluble human ACE2 (angiotensin-converting enzyme 2) have been previously described [9,10,11,12]. Spike (S) mutations of SARS-CoV-2 lineage B.1.620 (GISAID Accession ID: EPI_ISL_1540680) and R.1 (GISAID Accession ID: EPI_ISL_3183767) were introduced into the expression plasmid for the S protein of SARS-CoV-2 B.1 by hybrid PCR using overlapping primers. PCR products purified from an agarose gel (NucleoSpin Gel and PCR Clean-up, Macherey-Nagel, Düren, Germany) were mixed and subjected to PCR with primers corresponding to the 3′ and 5′ ends full-length S protein sequence. Generated open reading frames were ligated with linearized pCG1 plasmid (kindly provided by Roberto Cattaneo, Mayo Clinic College of Medicine, Rochester, MN, USA). All S protein sequences were verified by sequencing (Microsynth SeqLab, Göttingen, Germany).

### 2.3. Production of Pseudotype Particles

Production of rhabdoviral pseudotypes bearing SARS-CoV-2 spike protein has been previously described [13]. In brief, 293T cells were transfected with expression plasmid for SARS-CoV-2 S protein, VSV-G or control plasmid by calcium-phosphate precipitation. At 24 h posttransfection, cells were inoculated with VSV*ΔG-FLuc [14], a replication-deficient vesicular stomatitis virus that lacks the genetic information for VSV-G and instead codes for two reporter proteins, enhanced green fluorescent protein (eGFP) and firefly luciferase (FLuc) (kindly provided by Gert Zimmer) at a multiplicity of infection of 3. Following 1 h incubation, the inoculum was removed, and cells were washed with phosphate-buffered saline (PBS). Subsequently, cells received culture medium containing anti-VSV-G antibody (culture supernatant from I1-hybridoma cells; ATCC no. CRL-2700; except for cells expressing VSV-G, which received only medium) to neutralize residual input virus. After 16–18 h, the culture supernatant was harvested, separated from cellular debris by centrifugation for 10 min at 4000× *g* at room temperature, and the clarified supernatants were stored at −80 °C. 

### 2.4. Analysis of Spike Protein-Mediated Cell Entry

For cell entry study, target cells were seeded in 96-well plates. At 20 h post seeding, the cells were inoculated with equal volumes of pseudotype particles. At 18 h postinoculation, pseudotype entry efficiency was quantified by measuring the activity of virus-encoded luciferase. For this, cells were lysed using PBS containing 0.5% triton X-100 (Carl Roth, Karlsruhe, Germany) for 30 min at RT. Afterwards, cell lysates were transferred into white 96-well plates and mixed with luciferase substrate (Beetle-Juice, PJK, Kleinblittersdorf, Germany) before luminescence was measured using a Hidex Sense Plate luminometer (Hidex, Turku, Finland).

### 2.5. Production of Soluble ACE2

The production of soluble human ACE2 equipped with the Fc-portion of human immunoglobulin G at the C-terminus (solACE2-Fc) has been described in detail previously [15]. Briefly, 293T cells were seeded and transfected with expression plasmid for soluble hACE2. After overnight incubation, the medium was replaced, and the cells further incubated for 38 h before the supernatant was collected and centrifuged. The clarified supernatant was concentrated (100×) using a Vivaspin protein concentrator column (molecular weight cut-off of 30 kDa; Sartorius, Göttingen, Germany). The concentrated soluble ACE2 was stored at −80 °C.

### 2.6. Analysis of ACE2 Binding by Flow Cytometry

In order to test the binding of the different S proteins to ACE2, 293T cells were seeded in 6-well plates and transfected with expression plasmid for the respective SARS-CoV-2 S protein by calcium-phosphate precipitation. Cells transfected with empty plasmid served as a negative control. At 24 h posttransfection, the medium was replaced. At 48 h posttransfection, the culture medium was removed, cells were resuspended in PBS, transferred into 1.5 mL reaction tubes and pelleted by centrifugation. All centrifugation steps were carried out at room temperature at 600× *g* for 5 min. Subsequently, the supernatant was aspirated and the cells were washed with PBS containing 1% bovine serum albumin (BSA, PBS-B) and pelleted by centrifugation. Next, cell pellets were resuspended in 250 µL PBS-B containing soluble hACE2-Fc (1:200) and rotated at 4 °C for 60 min using a Rotospin rotator disk (IKA). Then, cells were pelleted, washed and resuspended in 250 µL PBS-B containing anti-human AlexaFlour-488-conjugated antibody (1:200; Thermo Fisher Scientific) and rotated again for 60 min at 4 °C. Finally, the cells were washed with PBS-B, resuspended in 100 µL PBS-B and subjected to flow cytometric analysis using an ID7000 Spectral Cell Analyzer (Sony Biotechnology, San Jose, CA, USA). Median channel fluorescence data were further analyzed using the ID7000 software.

### 2.7. Collection of Serum and Plasma Samples

Healthcare professionals vaccinated with either two doses of the mRNA vaccine BNT162b2 (BNT) or a first dose of the vectored vaccine AZD1222 (AZ) followed by a second dose of BNT were recruited as part of prospective studies investigating seroconversion within the healthcare system (e.g., CoCo (COVID-19 Contact) study, https://www.cocostudie.de/, accessed on 1 October 2022). Specific details on the samples can be found in Table S1. Serum samples were heat-inactivated at 56 °C for 30 min prior to neutralization experiments.

### 2.8. Neutralization Assay

For neutralization assay, S protein bearing pseudotype particles were pre-incubated at 37 °C for 30 min in the presence of different concentrations of monoclonal antibody (Casirivimab, Imdevimab, Bamlanivimab, Etesevimab, Sotrovimab or an unrelated human control IgG) (concentration spectrum: from 10 to 10–5 µg/mL). Alternatively, pseudotype particles were pre-incubated in the presence of different concentrations of plasma or serum from vaccinated individuals (diluted from 1:25 to 1:6400). Following incubation, mixtures were inoculated onto Vero cells. Pseudotype particles incubated with medium served as controls. Transduction efficiency was determined at 16–18 h postinoculation as described above.

### 2.9. Data Analysis

Data analysis was carried out using Microsoft Excel (as part of Microsoft Office Professional Plus, version 2016, Microsoft Corporation) and GraphPad Prism version 8.3.0 (GraphPad Software). Statistical significance was assessed using either one-way ANOVA with Dunnett’s post hoc test (data on S protein particle incorporation and cleavage, ACE2 binding, and S protein-driven cell entry) or the Friedman test with Dunn’s comparisons test (neutralization data). Only p-values of 0.05 or lower were considered statistically significant (*p* > 0.05, not significant (ns); *p* ≤ 0.05, *; *p* ≤ 0.01, **; *p* ≤ 0.001, ***). In order to calculate the serum/plasma dilutions that result in half-maximal inhibition of S protein-driven cell entry (neutralizing titer 50, NT50), a non-linear regression model was used.

## 3. Results

### 3.1. The Spike Proteins of SARS-CoV-2 Lineages B.1.620 and R.1 Differ Greatly with Respect to the Number of Mutations

**Figure 1:**
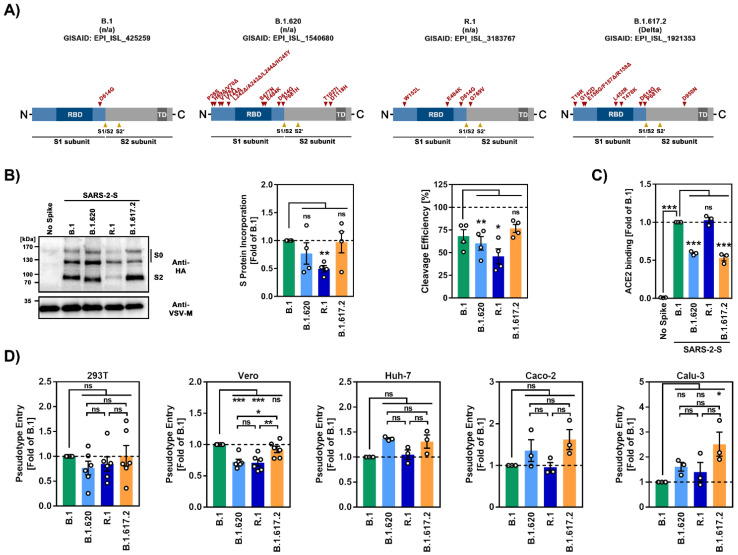
Spike proteins of SARS-CoV-2 lineages B.1.620 and R.1 differ regarding cleavability, particle incorporation and ACE2 binding compared to the spike protein of the SARS-CoV-2 B.1 lineage. (**A**) Schematic overview of the S protein domain organization of SARS-CoV-2 lineages B.1, B.1.620, R.1 and B.1.617.2. The location of mutations compared to the S protein of the original virus (Wuhan-Hu-01 isolate) is shown. RBD, receptor-binding domain; TD, transmembrane domain; S1/S2 and S2′, cleavage sites for host cell proteases. (**B**) Particle incorporation of SARS-CoV-2 S proteins. The incorporation of S proteins into VSV (vesicular stomatitis virus) pseudotypes was analyzed by immunoblot using an antibody against a C-terminal hemagglutinin (HA) tag (left panel). Bands corresponding to uncleaved precursor SARS-CoV-2 S protein (S0) and S2 subunit are labeled. Detection of VSV-M was used as loading control. A representative blot is shown, and similar results were obtained in four independent experiments. Total (mean) levels of SARS-CoV-2 S protein in particles were quantified with respect to the corresponding VSV-M signals and subsequently normalized (B.1 = 1, middle panel). Further, cleavage efficiency for each S protein was quantified (right panel). For this, total S protein signals were set as 100% and the relative percentage of S0 and S2 signals was determined. The mean from four independent experiments is shown. Error bars indicate the standard error of the mean (SEM). Statistical significance of differences between WT and variant S proteins was analyzed by one-way analysis of variance (ANOVA) with Dunnett’s post hoc test (*p* > 0.05, not significant (ns); *p* ≤ 0.05, *; *p* ≤ 0.01, **). (**C**) Strong ACE2 binding of R.1 S protein. Transfected 293T cells expressing the indicated S proteins were incubated with soluble ACE2 containing a C-terminal Fc-tag. Subsequently, the cells were stained with anti-human AlexaFlour-488-conjugated secondary antibody and subjected to flow cytometric analysis. Cells transfected with empty plasmid served as negative control and ACE2 binding was normalized against B.1 (=1). The mean data of three biological replicates is shown, error bars represent the SEM. The statistical significance of differences between WT and variant S proteins was analyzed by one-way ANOVA with Dunnett’s post hoc test (*p* > 0.05, ns; *p* ≤ 0.001, ***). (**D**) B.1.620 and R.1 S proteins drive efficient entry into human cell lines. Particles pseudotyped with the indicated S proteins were inoculated onto four different human cell lines (293T, Huh-7, Caco-2, Calu-3) and one African green monkey cell line (Vero). Transduction efficiency was quantified by measuring virus-encoded luciferase activity in cell lysates at 16–18 h post transduction. Presented are the mean data from three to six biological replicates (each conducted with technical quadruplicates) for which transduction was normalized against B.1 (=1). Error bars indicate the SEM. Statistical significance of differences between was analyzed by one-way ANOVA with Dunnett’s post hoc test (*p* > 0.05, ns; *p* ≤ 0.05, *; *p* ≤ 0.01, **; *p* ≤ 0.001, ***; please see also Figure S1B).

The SARS-CoV-2 lineage B.1.620 was first observed in western and central Africa (earliest sequences in the GISAID (Global Initiative on Sharing All Influenza Data) database were reported from Senegal, Cameroon and the Central African Republic) and dispersed into neighboring countries, Asia, Europe, and North and Central America in early to mid-2021 (Figure S1A). It carries a unique combination of mutations in the S protein [16] (Figure 1A), some of which have also been observed in the variants of concern (VOC) B.1.1.7 (Alpha), B.1.351 (Beta), P.1 (Gamma) and B.1.1.529 (Omicron). The N-terminal domain (NTD) of the S protein, which contains an antigenic supersite [17,18,19,20], is heavily mutated in the B.1.620 lineage, and the mutations may reduce binding of neutralizing antibodies. Furthermore, mutations S477N and E484K, which are located in the receptor binding domain (RBD) (Figure 1A), might modulate ACE2 interactions [21,22] and reduce neutralization sensitivity to RBD-specific antibodies [23,24,25,26]. Finally the B.1.620 spike protein contains mutation D614G, which is associated with increased transmissibility [27,28], and mutation P681H, which is located at the N-terminus of the S1/S2 cleavage site but does not increase spike protein cleavage by furin [29,30]. In the first half of 2021, the SARS-CoV-2 lineage R.1 (sublineage of B.1.1.316) spread to at least 30 countries, with the majority of cases observed in Japan and the US [31,32] (Figure S1A). In Japan, its prevalence reached 40% [33], but after a short period of expansion, infections declined and R.1 was replaced by the B.1.1.7 and B.1.617.2 lineages [31,32,34]. In contrast to the B.1.620 S protein, its counterpart in R.1 is not heavily mutated. It harbors the E484K and D614G mutations described above, as well as one mutation in the NTD (W152L), which is believed to be associated with evasion of neutralizing antibodies [35,36], and one mutation in the in the S2 subunit (G769V) (Figure 1A).

### 3.2. The Spike Proteins of SARS-CoV-2 Lineages B.1.620 and R.1 Differ Regarding Cleavability, Particle Incorporation and ACE2 Binding Compared to the Spike Protein of the SARS-CoV-2 B.1 Lineage

We investigated host cell entry of B.1.620 and R.1 and its inhibition using rhabdoviral reporter particles pseudotyped with the respective S proteins, which are an adequate and well-established model for SARS-CoV-2 entry into cells and is inhibition by neutralizing antibodies [37]. The S proteins of B.1 (identical to the S protein of the Wuhan-Hu-1 isolate, except for the presence of mutation D614G), which circulated early in the pandemic, and B.1.617.2 (Delta variant) served as controls. Immunoblot analyses revealed that the S proteins of B.1.620 and R.1 were robustly incorporated into particles (Figure 1B), although incorporation of R.1 S protein was reduced as compared to the other S proteins studied. All S proteins were cleaved at the S1/S2 site, as expected. Cleavage efficiency of B.1.6120 and particularly R.1 S proteins was reduced, while cleavage of B.1.617.2 S protein was augmented (although this effect was not statistically significant) relative to B.1. spike (Figure 1B), in keeping with the published data [11]. Further, the S2 band of B.1.620 migrated slightly faster as compared to the other S2 bands (Figure 1B) and the underlying reasons are at present unclear. Binding of S protein expressing cells to ACE2 fused to the Fc portion of human immunoglobulin G revealed strong ACE2 binding to R.1 S protein, while binding to B.1.620 and B.1.617.2 S proteins was reduced as compared to B.1 S protein (Figure 1C). Next, we analyzed host cell entry mediated by the B.1.620 and R.1 S proteins. For this, we employed the human cell lines 293T (kidney), Huh-7 (liver), Caco-2 (colon) and Calu-3 (lung) and the African green monkey cell line Vero (kidney) as targets. The B.1.620 and R.1 S proteins mediated entry into 293T, Huh-7, Caco-2 and Calu-3 cells with similar efficiency as the B.1 S protein, while entry into Vero cells was significantly less efficient (Figure 1D and Figure S1B). Further, Calu-3 cell entry driven by the B.1.617.2 S protein was enhanced relative to B.1 S protein, in agreement with published data [11,38,39] (Figure 1D).

### 3.3. The Spike Proteins of SARS-CoV-2 Lineages B.1.620 and R.1 Display Reduced Sensitivity to Neutralization by Antibodies Induced upon Vaccination and Clinically-Used Monoclonal Antibodies

**Figure 2:**
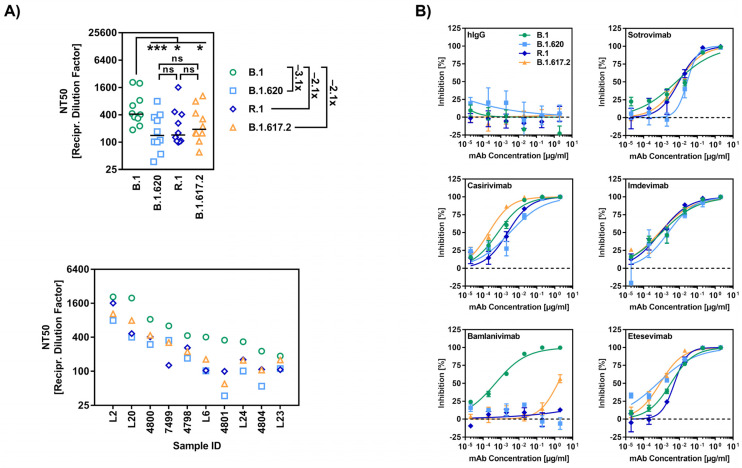
SARS-CoV-2 lineages B.1.620 and R.1 evade antibody-mediated neutralization. (**A**,**B**) The S proteins of SARS-CoV-2 B.1.620 and R.1 evade neutralization by antibodies induced by vaccination or employed for COVID-19 therapy. S protein-bearing particles were incubated at 37 °C for 30 min in the presence of the indicated plasma samples from BNT/BNT or AZ/BNT vaccinated individuals (panel (**A**)) or therapeutic monoclonal antibodies (panel (**B**)) before being inoculated onto Vero cells. Transduction efficiency was quantified as stated for Figure 1D and used to calculate the plasma dilution factor that leads to a 50% reduction in transduction (NT50, panel (**A**)). Data for ten serum samples from vaccinated donors are presented. Black lines indicate the median and numbers on the right represent the fold change in NT50 compared to B.1. Statistical significance of differences between individual groups was analyzed by Friedman test with Dunn’s multiple comparisons test (panel (**A**); *p* > 0.05, ns; *p* ≤ 0.05, *; *p* ≤ 0.001, ***; please see also Figure S1C).

We next studied susceptibility of the B.1.620 and R.1 S proteins to antibody-mediated neutralization, employing plasma and/or serum from individuals who had either received two immunizations with the mRNA vaccine BNT162b2 (BNT), or a first dose of the vectored vaccine AZD1222 (AZ) followed by a second dose of BNT (Table S1), which represented widely used vaccination regimens in Germany at the time when B.1.620 and R.1 circulated. Neutralization of particles bearing the B.1.620 and R.1 S proteins was 3.1- and 2.1-fold, respectively, less efficient than that of particles bearing the B.1 S protein, and was comparable to that measured for particles bearing the B.1.617.2 S protein (Figure 2A). Finally, we analyzed inhibition of the B.1.620 and R.1 S proteins by monoclonal antibodies used for COVID-19 therapy (Figure 2B). Four out of five antibodies inhibited all S proteins tested efficiently and to roughly comparable levels. In contrast, all S proteins with the exception of the B.1 S protein were largely fully resistant against Bamlanivimab (Figure 2B), which is line with previous reports on B.1.617.2 [40,41] or the presence of mutation E484K in the case of B.1.620 and R.1 [42].

## 4. Discussion

We observed that the S proteins of B.1.620 and R.1 drive robust cell entry into various cell lines and evade antibody-mediated neutralization with similar efficiency as the B.1.617.2 S protein. Some of our observations are noteworthy:

Cleavage of the B.1.620 and particularly R.1 S proteins was less efficient, while cleavage of the B.1.617.2 S protein was slightly more efficient (not statistically significant) than the B.1. S protein. The latter phenotype might result from the presence of the P681R mutation in the B.1.617.2 S protein, which is located within the S1/S2 site and increases cleavability, transmissibility and pathogenicity [38,39]. Why the S2 band of B.1.620 S protein migrated faster during gel electrophoresis is at present unclear. However, the faster migration might reflect cleavage at a site different from the canonical S1/S2 site or altered posttranslational modifications.

The finding that binding of B.1.620 S protein to soluble ACE2 was less efficient as compared to B.1 S protein is somewhat surprising as it has been previously reported that RBD mutation S477N strengthens ACE2 binding [21], whereas RBD mutation E484K slightly reduces ACE2 interaction [43]. However, in the context of the Omicron S protein, it has also been shown that the combination of several RBD mutations that reduce ACE2 interaction with some RBD mutations that strengthen ACE2 interaction can result in an overall increase of ACE2 binding [44], and the opposite trend might be true for the combination of RBD mutations S477N and E484K. The R.1 S protein bound to ACE2 with higher efficiency as compared to the B.1 S protein, despite harboring RBD mutation E484K that is associated with a subtle reduction in ACE2 interaction efficiency [43]. Here, one can speculate that the reduced cleavage phenotype of the R.1 S protein compared to the B.1 S protein might restrict the conformational flexibility of the R.1 S protein and thus may favor a conformation required for efficient ACE2 binding. While we did not specifically test this hypothesis, it should be noted that Zhang and colleagues made a similar observation when they compared ACE2 binding of the S protein of an early SARS-CoV-2 isolate (without D614G mutation) and a mutant version thereof that contained an altered S1/S2 cleavage site and therefore was not cleaved by furin [45]. In addition, we note that ACE2 binding to B.1.617.2 S protein was less efficient than the B.1 S protein, although in a previous study we detected comparable binding [11]; subtle differences in experimental conditions might be responsible. Finally, it should be stated that staining with the neutralizing antibody Imdevimab and subsequent FACS analysis revealed robust expression of all S proteins at the cell surface (Figure S2), indicating that differences in ACE2 binding were not due to differences in S protein surface expression.

Regarding host cell entry, B.1.620 and R.1 S proteins did not mediate increased entry into any of the cell lines tested as compared to B.1 spike. In contrast, the B.1.617.2 S protein facilitated entry into Calu-3 lung cells with higher efficiency than the B.1 S protein, in keeping with our published data [11], and this phenotype was most likely due to mutation P681R. Thus, P681R increases S protein cleavage at the S1/S2 site [38,39], which is a prerequisite for S protein activation by TMPRSS2 and Calu-3 cell entry [46].

The observation that the S proteins of B.1.620 and R.1 were resistant against the therapeutic antibody Bamlanivimab is not surprising given that mutation E484K, which is present in the RBDs of both S proteins, is located in the Bamlanivimab epitope and confers Bamlanivimab resistance [47]. Further, the evasion of vaccination-induced neutralizing antibodies by B.1.620 does not come as a surprise, considering that this lineage harbors several mutations in the NTD and RBD, some of which are known to reduce antibody-mediated neutralization. In contrast, the observation that R.1 S protein evaded antibody-mediated neutralization with similar efficiency as B.1.617.2 S protein was surprising since R.1 S harbors only two additional mutations in the S1 subunit of the S protein, W152L and E484K, compared to B.1 S. The role of E484K in evasion of neutralizing antibodies is well established [26]. However, the robust evasion of neutralization by antibodies induced by BNT/BNT or AZ/BNT vaccination suggests a substantial contribution of W152L, which has also been suggested by other studies [35,36], although functional data are so far missing. Collection of serum/plasma from BNT/BNT-vaccinated individuals was carried out within one month after the second vaccination, while samples from AZ/BNT-vaccinated individuals were taken within two to four months after the second vaccination. While the discrepancy in sampling time between BNT/BNT- and AZ/BNT-vaccinated groups constitutes a limitation of this study, we did not observe differences in the extent of immune evasion by B.1.617.2, B.1.620 and R.1 for the two vaccination groups.

Collectively, our results, which await confirmation with authentic virus, suggest that B.1.620 and R.1 evade neutralizing antibodies with similar efficiency as B.1.617.2, and should thus be able to spread in an immunologically non-naïve target population.

## Data Availability

The data presented in this study are available on request from the corresponding authors.

## Supplementary Materials

The following supporting information can be downloaded at: https://www.mdpi.com/article/10.3390/v14112475/s1, Figure S1: Epidemiology, cell entry and neutralization of SARS-CoV-2 lineages B.1.620 and R.1; Figure S2: Surface expression of SARS-CoV-2 lineage B.1.620 and R.1 spike proteins; Table S1: Information on vaccinee sera.
