# Supplementary material for: Host Cell Entry and Neutralization Sensitivity of SARS-CoV-2 Lineages B.1.620 and R.1
Source: Viruses. 2022 Nov 9;14(11):2475. doi: 10.3390/v14112475 (PMC9698971; doi:10.3390/v14112475)
Supplement: Supplementary file 1 [file viruses-14-02475-s001.zip › viruses-1996992-supplementary.pdf]

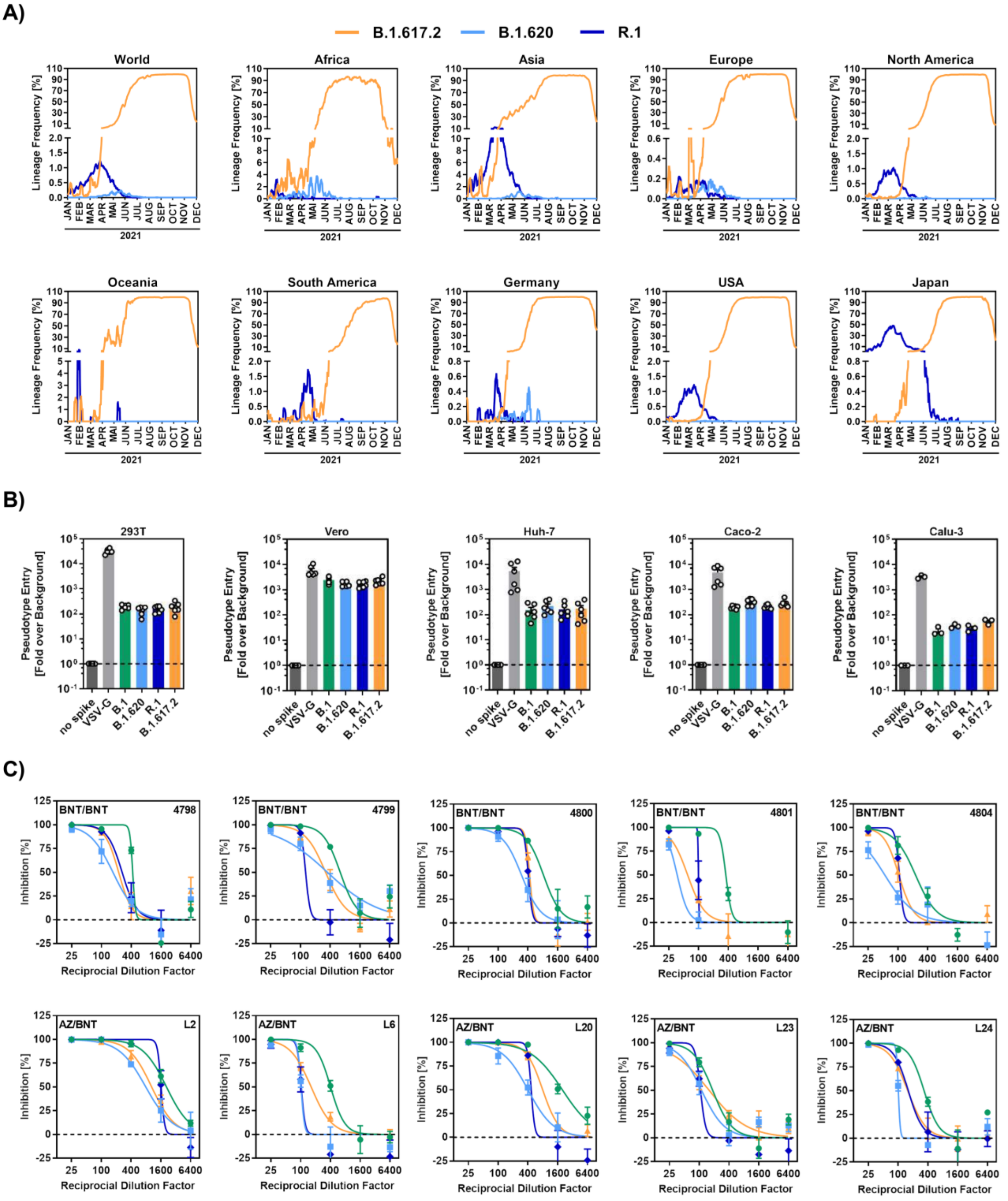

Figure S1. Epidemiology, cell entry and neutralization of SARS-CoV-2 lineages B.1.620 and R.1. A) Epidemiology of SARS-CoV-2 lineages in 2021. Relative frequency of SARS-CoV-2 lineages B.1.617.2, B.1.620 and R.1 in 2021 on global, continent and country level was calculated based on submitted sequences to GISAID (Global Initiative on Sharing All Influenza Data; https://gisaid.org/) database. Data were retrieved from the CoV-Spectrum database (https://cov-spectrum.org/). B.1.617.2, B.1.620 and R.1 main and sublineages were included in the analysis. B) Data on pseudovirus entry (Fig. 1D) were normalized against the assay background (luciferase activity obtained of target cells inoculated with particles containing no viral surface protein, set as 1). Further, data on cell entry by pseudoviruses bearing the glycoprotein of vesicular stomatitis virus (VSV-G) are shown. C) Individual neutralization data for

each plasma sample. For normalization, entry of pseudoviruses in the absence of plasma was set as 0% inhibition (indicated by the dashed line).

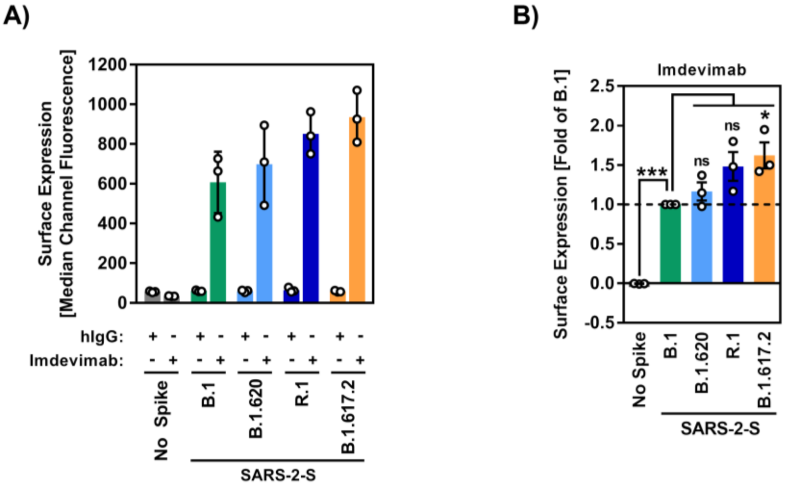

Figure S2. : Surface expression of SARS-CoV-2 lineage B.1.620 and R.1 spike proteins. A) 293T cells expressing the indicated S proteins were incubated with either control human IgG (hIgG) or Imdevimab at a concentration of 1 µg/ml, followed by staining with anti-human AlexaFlour-488-conjugated secondary antibody and subsequently subjected to flow cytometric analysis. Cells transfected with empty plasmid served as negative control. The mean data of three biological replicates are shown (median channel fluorescence), error bars represent the standard deviation. B) The data on Imdevimab binding were normalized against B.1 (=1). The mean data of three biological replicates is shown (fold change in median channel fluorescence), error bars represent the SEM. Statistical significance was analyzed by one-way ANOVA with Dunnett's post-hoc test ($p > 0.05$, ns; $p \leq 0.05$, *; $p \leq 0.001$, ***).

Table S1. Information on vaccinee sera.

Sample ID	Sex	Age group (years)	SARS-CoV-2 Infection	Vaccination	Time between First and Second Vaccine Dose (days)	Time between Last Vaccination and Sampling (days)
L02	Male	25-34	No	AZD1222/BNT162b2	80	65
L06	Female	25-34	No	AZD1222/BNT162b2	80	65
L15	Male	25-34	No	AZD1222/BNT162b2	70	92
L20	Female	18-24	No	AZD1222/BNT162b2	84	81
L23	Female	25-34	No	AZD1222/BNT162b2	84	98
L24	Female	18-24	No	AZD1222/BNT162b2	84	98
4801	Female	25-34	No	BNT162b2/BNT162b2	21	18
4799	Male	25-34	No	BNT162b2/BNT162b2	19	21
4797	Male	35-44	No	BNT162b2/BNT162b2	23	22
4798	Male	45-54	No	BNT162b2/BNT162b2	21	21
4800	Female	45-54	No	BNT162b2/BNT162b2	22	21
4804	Female	55-64	No	BNT162b2/BNT162b2	26	21
